# Assessing the Utility of Hydrogen, Carbon and Nitrogen Stable Isotopes in Estimating Consumer Allochthony in Two Shallow Eutrophic Lakes

**DOI:** 10.1371/journal.pone.0155562

**Published:** 2016-05-11

**Authors:** Jari Syväranta, Kristin Scharnweber, Mario Brauns, Sabine Hilt, Thomas Mehner

**Affiliations:** 1 Department of Fish Biology and Ecology, Leibniz-Institute of Freshwater Biology and Inland Fisheries, Berlin, Germany; 2 Lake Ecology Section, Department of Bioscience, Aarhus University, Silkeborg, Denmark; 3 Evolutionary Biology Centre, Department of Ecology and Genetics; Limnology, Uppsala University, Uppsala, Sweden; 4 Department of River Ecology, Helmholtz Centre for Environmental Research -UFZ, Magdeburg, Germany; 5 Department of Ecosystem Research, Leibniz-Institute of Freshwater Biology and Inland Fisheries, Berlin, Germany; Center for Nanosciences and Nanotechnology, MEXICO

## Abstract

Hydrogen stable isotopes (δ^2^H) have recently been used to complement δ^13^C and δ^15^N in food web studies due to their potentially greater power to separate sources of organic matter in aquatic food webs. However, uncertainties remain regarding the use of δ^2^H, since little is known about the potential variation in the amount of exchangeable hydrogen (*H*_*ex*_) among common sample materials or the patterns of δ^2^H when entire food webs are considered. We assessed differences in *H*_*ex*_ among the typical sample materials in freshwater studies and used δ^2^H, δ^13^C and δ^15^N to compare their effectiveness in tracing allochthonous matter in food webs of two small temperate lakes. Our results showed higher average amounts of *H*_*ex*_ in animal tissues (27% in fish and macroinvertebrates, 19% in zooplankton) compared to most plant material (15% in terrestrial plants and 8% in seston/periphyton), with the exception of aquatic vascular plants (23%, referred to as macrophytes). The amount of *H*_*ex*_ correlated strongly with sample lipid content (inferred from C:N ratios) in fish and zooplankton samples. Overall, the three isotopes provided good separation of sources (seston, periphyton, macrophytes and allochthonous organic matter), particularly the δ^2^H followed by δ^13^C. Aquatic macrophytes revealed unexpectedly high δ^2^H values, having more elevated δ^2^H values than terrestrial organic matter with direct implications for estimating consumer allochthony. Organic matter from macrophytes significantly contributed to the food webs in both lakes highlighting the need to include macrophytes as a potential source when using stable isotopes to estimate trophic structures and contributions from allochthonous sources.

## Introduction

Stable isotope analysis (SIA) is now routinely used to study food webs and energy flow within aquatic ecosystems. Carbon isotope ratios (δ^13^C) are often used as tracers of sources of energy fueling the food webs, while nitrogen isotope ratios (δ^15^N) are mainly used to quantify trophic levels of organisms and sources of inorganic nitrogen [1]. SIA studies have provided invaluable information on the contributions of allochthonous resources [2–6] and methane-derived carbon to animal consumers in freshwater food webs [7–10], both features that would be difficult or often impossible to study using more conventional methods, such as gut content analyses. However, many studies are faced with substantial difficulties in separating potential sources with sufficient precision due to their overlapping δ^13^C and δ^15^N values and/or too many sources to be separated with only two isotopes. In some cases, enrichment studies, using for example ^13^C-bicarbonate [4, 11–12] or C_4_ plant material such as cane sugar or maize (*Zea mays*) [13–15], are a useful way to alter the naturally occurring isotope ratios within the sources to enhance separation. Unfortunately, these methods are not always applicable due to high costs and effort, and alternative measures are needed.

Following recent advances in methodological issues [16–17], stable hydrogen isotopes (δ^2^H or δD) have now become well established tracers, for example in studies of animal migrations, particularly in terrestrial systems [18–19]. Doucett et al. [20] proposed δ^2^H as a potential alternative to δ^13^C in tracing allochthonous organic matter contributions in aquatic food webs by showing that the δ^2^H provided a greater separation between terrestrial and aquatic production in arid lotic systems. This stems from the fact that δ^2^H values in terrestrial plants are considerably elevated due to additional discrimination during the transpiration [21], which is not observed in aquatic plants. Differences in δ^2^H values between terrestrial and aquatic production can exceed 100‰, particularly in arid environments [20], but substantial separation is also possible in more mesic environments [22–23]. Therefore, δ^2^H values are currently widely employed in many aquatic food web studies, but particularly in tracing allochthonous contributions in freshwater ecosystems [23–27].

However, the use of δ^2^H has several caveats that can reduce its robustness in food web studies. Over 20% of the H bound in organic sample material can be freely exchangeable with H in ambient water vapor [16]. While the issue of exchangeable H (*H*_*ex*_) in analytical procedures can now efficiently be controlled for, there is still substantial lack of knowledge about differences in the amount of *H*_*ex*_ among sample materials. The amount of *H*_*ex*_ in some tissues, like keratin, is rather well known and consistent [17] but less is known about the fraction in many other tissues, even though these are continuously used in δ^2^H studies. This uncertainty in the amount of *H*_*ex*_ potentially has serious implications for sample treatment, standardization and results interpretation [28]. Consumers also acquire part of their tissue H from dietary/environmental water, as shown in several controlled experiments [29–33], which complicates inferences of the origin of organic matter supporting consumers. Particular uncertainties remain on the influence of environmental water when whole food webs and multiple trophic levels are considered, as the influence of environmental H may multiply at higher trophic levels. In fact, studies using δ^2^H on an entire food web scale are still very rare. Furthermore, there is considerable debate on whether and to what extent discrimination in δ^2^H values occurs during trophic transfers [18, 22, 30–31]. Discrimination likely results from the increased intake of environmental water but can potentially be masked by the substantial influence of lipids on tissue δ^2^H values. Lipids are highly depleted in ^2^H [34–35] and may have δ^2^H values as much as 100‰ lower compared to other body tissues [18, 32], having a substantial influence on bulk tissue δ^2^H values in lipid-rich tissues or animals [22, 27, 32]. Also, lipids contain very little or no *H*_*ex*_ [16, 36] potentially complicating sample preparation procedures among samples with variable lipid contents.

Another potential concern arises from recent evidence of surprisingly elevated δ^2^H values in aquatic macrophytes [37–38]. The significance of macrophytes (aquatic vascular plants) as an energy source for aquatic consumers has long been debated [39–41], and strong evidence exists for their dietary role especially for benthic consumers [41–44]. Reported δ^2^H values of macrophytes have been considerably higher than those in algal (phytoplankton or attached algae) production in the same system, approaching the δ^2^H values analyzed from terrestrial plants. Such overlapping δ^2^H values between autochthonous macrophytes and allochthonous terrestrial primary producers might severely undermine the robustness of using H isotopes to trace the contributions of allochthonous matter in aquatic food webs, particularly in systems with potentially significant contribution of macrophyte-derived organic matter to consumers.

Here we first compared the amounts of *H*_*ex*_ among all our sample materials to assess whether the commonly used keratin standards are suitable for analyzes of δ^2^H of typical aquatic organisms. We then elucidated entire food web structures using stable isotopes of hydrogen, carbon and nitrogen and assessed their suitability in tracing the contributions from allochthonous organic matter in food webs of two small temperate lakes. We estimated allochthonous contributions to consumers using isotope mixing models and employed either δ^13^C and δ^15^N values or δ^13^C, δ^15^N and δ^2^H values to assess the added value of δ^2^H in temperate lowland lakes. In addition, we evaluated the importance of macrophytes in estimating allochthonous organic matter contributions by running mixing models with and without macrophytes as one potential source.

## Materials and Methods

Samples were collected from two eutrophic, shallow lakes located in North-Eastern Germany during summer 2010. Kleiner Gollinsee (hereafter referred to as Gollinsee) has an area of 3.3 ha, mean depth of 1.7 m, total phosphorus (TP) concentration of 42 μg l^-1^ and dissolved organic carbon (DOC) concentration of 12.3 mg l^-1^. Gollinsee is turbid (light attenuation: 1.2 ± 0.1 m^-1^) and dominated by phytoplankton whereas Schulzensee (3.9 ha, 2.3 m, TP 34 μg l^-1^ and DOC 11.3 mg l^-1^, respectively) has higher water clarity (light attenuation: 0.7 ± 0.1 m^-1^) and approximately 22% of the lake area is colonized by submerged macrophytes (*Ceratophyllum submersum*) [45]. Both lakes are surrounded by alder trees (*Alnus glutinosa*) and reed stands (*Phragmites australis*), the latter contributing significantly to the C influx of both lakes [44]. Floating-leaved macrophytes (*Nymphaea alba* and *Nuphar lutea*) are growing in both lakes and cover 3% of the lake area in Gollinsee and 12% in Schulzensee [46]. Roach (*Rutilus rutilus*) was the dominant fish species in both lakes accounting for almost two-thirds of fish biomass, while sunbleak (*Leucaspius delineatus*) was highly abundant only in the turbid lake after a partial winterkill [47].

Aquatic plant samples (phytoplankton/seston, attached algae and macrophytes, *n* = 13) and terrestrial plant material (*n* = 6) were collected in June 2010 to analyze H, C and N stable isotope values in potential sources of organic matter to the lake food webs. Seston was collected by hauling a small mesh size (30 μm) net slowly behind the boat, picking out all larger zooplankton and detritus, diluting the cleaned sample into larger volume of water followed by further separation by sedimentation and drying in an oven. Periphyton was collected by scraping samples from floating and submerged macrophytes and from artificial polypropylene slides submersed into the lake. Periphyton samples were then picked clean from all visible animal and detritus particles, and dried in an oven. Aquatic macrophyte samples (*C*. *submersum*, *N*. *alba* and reed) were collected by dissecting fresh leaves from the plants which were then wiped clean and cut into smaller pieces, and dried in an oven. Samples from allochthonous organic matter (allo-om) were taken from birch (*Betula pendula*) and alder trees and included fresh and conditioned leaves from the lake littoral (detritus). Fresh and conditioned samples were analyzed separately but did not differ in their isotope values; hence they were all pooled to represent *allo-om*.

Zooplankton samples (*n* = 19) were collected three times (April, June and September 2010) during summer with several vertical hauls using zooplankton nets (mesh sizes 100 and 55μm) and replicate samples were collected during each sampling period from different sites. Samples were then brought to the laboratory and left overnight in clean tap water to allow gut evacuation. The next day these samples were identified, sorted and dried in an oven for later preparation for SIA. All zooplankton isotope values reported here represent samples of Cladocera comprising almost entirely of Daphniidae and Bosminidae. Benthic macroinvertebrate samples (*n* = 33) were collected in spring and summer with sweep-nets from depths of 0.5 − 1.0 m, identified visually to either family or genus level, sorted and placed into plastic jars with clean tap water for gut evacuation overnight.

Samples (*n* = 72) of fish, mainly roach, rudd (*Scardinius erythrophthalmus*), sunbleak, perch (*Perca fluviatilis*) and pike (*Esox lucius*) were collected throughout the summer with Nordic multi-mesh gillnets. In addition, young-of-the-year (YOY) cyprinid fish juveniles were collected with electrofishing in May. Fish were killed after capture and frozen for transport to the laboratory. Fishing was conducted by persons holding the official license for professional fishermen in Germany and hence followed the ethical standards and no further permits were needed. Access to and permission to fish in Schulzensee was provided by the Förderverein Feldberg-Uckermärkische Seen e.V. and by the Stiftung Pro Artenvielfalt in Gollinsee. Length and weight were measured and a small muscle sample was dissected from each individual fish. The muscle samples were then dried in an oven for later processing for SIA. In addition to all solid samples, four replicates of approximately 2 ml of lake water were sampled in June ~0.5 m below the lake surface from both lakes, and stored in 2 ml glass vials with air tight caps for later δ^2^H analysis of water.

All solid isotope samples were dried in an oven at 60°C and ground into homogenous powder using a mortar and pestle. A small subsample (0.6 mg) was then weighed into a tin cup for the analysis of δ^13^C and δ^15^N at the University of Jyväskylä, Finland, using a FlashEA 1112 elemental analyzer coupled to a Thermo Finnigan DELTAplus Advantage mass spectrometer (Thermo Electron Corporation, Waltham, MA, U.S.A.) following standard protocols and analytical precision better than 0.2‰ for both δ^13^C and δ^15^N. Two subsamples (0.4 mg) of each solid sample were also accurately weighed into silver cups for δ^2^H analyses. The two different sample sets for δ^2^H analysis were used to assess the amount of *H*_*ex*_ in different tissues types. Since significant part of the tissue H can be exchangeable [16], *H*_*ex*_ needs to be taken into account when analyzing δ^2^H from organic materials. Therefore one sample set was equilibrated in Colorado Plateau Stable Isotope Laboratory (CPSIL) ambient lab air with δ^2^H value -91 ± 8‰ (monthly average for given analysis month) and the other set under water vapor with a δ^2^H value +354 ± 1.3‰ following Wassenaar & Hobson [17]. The fraction of *H*_*ex*_ was then calculated from *f*(*H*_*ex*_) = (δ^2^H_sample1_—δ^2^H_sample2_) / (δ^2^H_water1_—δ^2^H_water2_), where sample1 and sample2 are the δ^2^H values for the same sample after equilibration in water vapor with two different δ^2^H values and water1 and water2 are the δ^2^H values of the water vapor [16, 36, 48–49].

All hydrogen isotope analyses from organic sample material were performed at CPSIL. Samples of organic materials were pyrolized to H_2_ and the isotope ratio was measured on the H_2_ gas [20]. Organic matter samples for δ^2^H were analyzed with a Thermo-Finnigan TC/EA and DeltaPLUS-XL (Thermo Electron Corporation, Bremen, Germany) using keratin, CBS (Caribou hoof) and KHS (Kudo horn) as normalization standards [16–17], and several secondary standards (Moose hair, Baleen BWB-II, Chitin TCI, Colorado River algae and IAEA-085). Hydrogen isotopes from water samples were analyzed by Laser Water Isotope Analyzer V2 (Los Gatos Research, Inc., Mountain View, CA, USA) at UC Davis Stable Isotope Facility. All values are reported in per mil notation (‰) and are in relation to the international standard of Vienna Standard Mean Oceanic Water (δ^2^H_VSMOW_). Analytical precision for δ^2^H of solid samples was < 3‰ and for water < 1‰.

We used R 2.15.2 [50] and Bayesian mixing models in the SIAR (Stable Isotope Analysis in R) package [51] to calculate the proportion of different carbon sources to consumer tissues. We calculated allochthonous organic matter contributions using both a two-isotope (CN, δ^13^C and δ^15^N) and a three-isotope model (HCN, δ^2^H, δ^13^C and δ^15^N) with periphyton, seston, allo-om and macrophytes as sources for primary consumers, and a HCN model with and without aquatic macrophytes as one potential source, to compare the results and the impact of using more isotopes and sources. While we were primarily interested in assessing the utility of hydrogen isotopes to trace the flow of allochthonous organic matter in aquatic food webs [20], we did not simply divide the potential energy sources into autochthonous and allochthonous origins but rather wanted to use all three isotopes to illustrate the structure of the entire food web in these lakes. For these models, we assumed no trophic discrimination for δ^2^H, but conservative 0.5 ± 0.2‰ for δ^13^C and 3.0 ± 0.5‰ discrimination for δ^15^N [52]. However, the H in environmental (or dietary) water directly influences consumer δ^2^H [18, 30], which needs to be accounted for in the model. We corrected for the dietary water using a model *ω*_compound_ = 1—(1—*ω*)^*t*^ from Solomon et al. [30], where *ω*_compound_ is the total H contribution from dietary water to consumer tissue δ^2^H at trophic level *t*, and *ω* is the proportion of tissue H derived from environmental water (see Wilkinson et al. [28] for a recent review on the potential effects of selecting suitable *ω*). We used *ω* = 0.173 from Solomon et al. [30] for all consumers and estimated trophic level *t* for each fish individual from a linear relationship between primary consumer (all invertebrates were assigned *t* = 2) δ^13^C and δ^15^N values in littoral and pelagic habitats following a model outlined in Karlsson and Byström [53]. The model first estimates the fraction of littoral carbon in a consumer (LF_cons_) from pelagic and littoral isotope baselines (here means of zooplankton/bivalves in pelagic and gastropods/isopods in littoral) from: LF_cons_ = [δ^13^C_cons_—δ^13^C_pel_−(δ^15^N_cons_—δ^15^N_pel_) × TS] ÷ (1—TS × BS) / (δ^13^C_lit_—δ^13^C_pel_), where TS is the slope of trophic discrimination for δ^13^C and δ^15^N (same values as for SIAR above) and BS is the slope of the linear relationship between the pelagic (pel) and littoral (lit) baselines. The trophic position of a consumer (*t*) can then be calculated from: *t* = 2 + ([δ^15^N_cons_—δ^15^N_pel_—(δ^13^C_lit_—δ^13^C_pel_) × BS × LF_cons_] / ΔN), where ΔN is the discrimination factor for δ^15^N. Fish were assumed to acquire allo-om indirectly through their prey and therefore mixing models of fish used invertebrates (for non-piscivorous fish) and invertebrates and prey fish (for piscivorous fish, i.e. large perch and pike) as prey sources. The estimates of allo-om contribution were then calculated using ratio calculation from each individual prey source [54, 55].

Differences in the amount of *H*_*ex*_ among sample types, and differences in mean allochthonous source contributions using either CN, HCN or no-macrophyte models, were examined using paired t-tests, after testing for normality of paired differences. In cases where the assumptions for normality and homoscedasticity were violated, we used a non-parametric Kruskal-Wallis test with pairwise comparisons. Multivariate analysis of variance (MANOVA) with effect size (partial *η*^*2*^) was used to assess how well the isotopes separated sources in these lakes and overall which isotope had the greatest separating power (strongest effect). Since lipids have less or negligible amount of *H*_*ex*_ compared to other tissues (e.g. [16, 36]), we used sample tissue C:N ratios as a proxy for lipid content [56–57] and examined the correlations between C:N ratio and the fraction of *H*_*ex*_ with Pearson’s correlation. All statistical tests were done using IBM SPSS Statistics 20.0 (IBM Company, Armonk, NY, U.S.A.)

## Results

**Fig 1:**
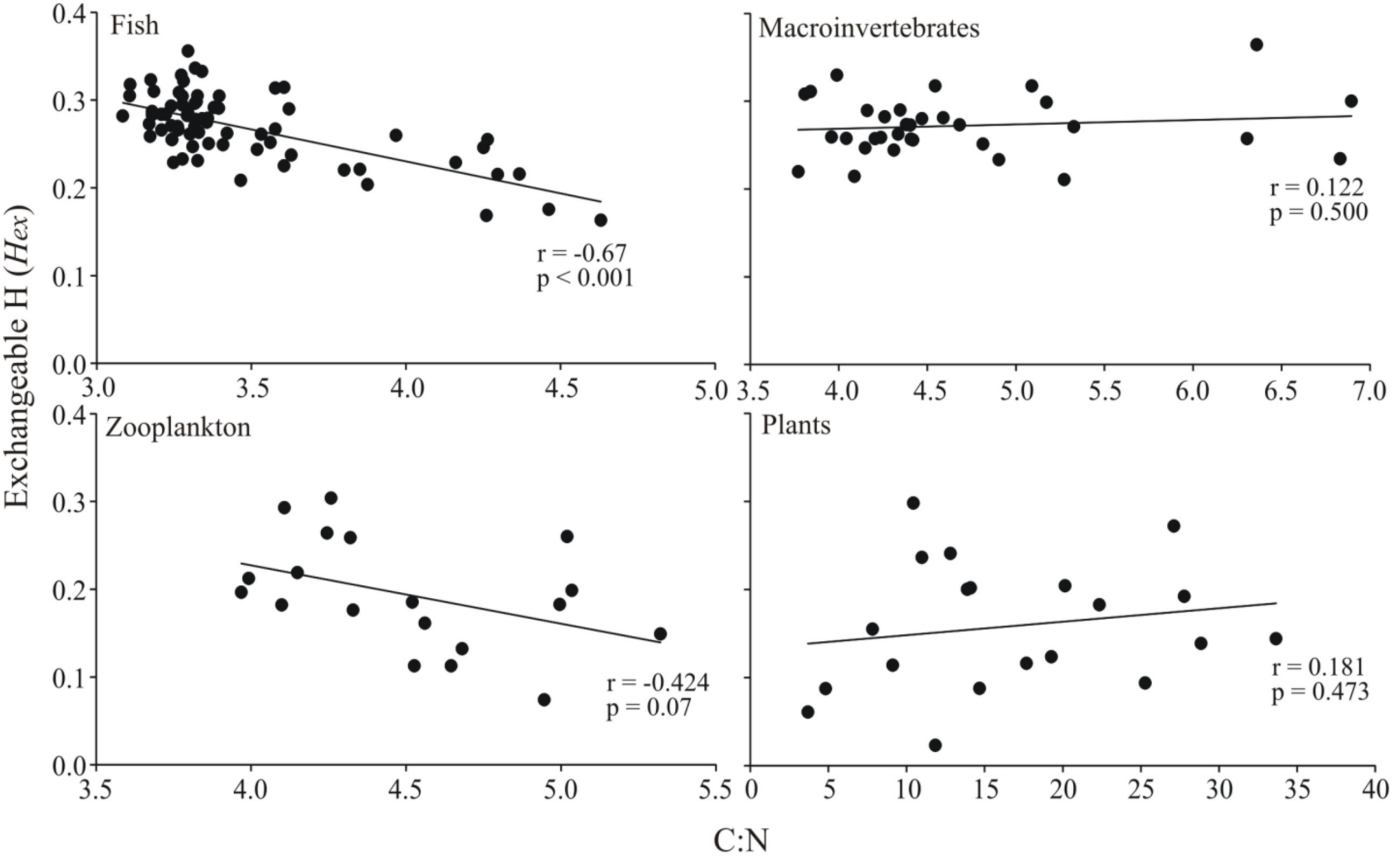
Correlation of *H*_*ex*_ and lipid content in sample tissues. Relationship between the fraction of exchangeable H (*H*_*ex*_) (0−1) and the C:N ratios (as proxy for lipid content) in sample materials. Values for Pearson’s correlation coefficients and p-values are given in each corresponding panel.

Fish and macroinvertebrates had consistently more *H*_*ex*_ in their tissues compared to zooplankton (*F*_*5*,*141*_ = 37.7, *p* < 0.001), while the amount in plant material was highly variable between aquatic and terrestrial plants (Table 1). Aquatic vascular plants (macrophytes) had significantly (*p* < 0.05, Table 1) more *H*_*ex*_ compared to both terrestrial plant material and seston/periphyton. There was a strong negative correlation (Pearson’s *r* = -0.67, *p* < 0.001, *n* = 72) between the amount of *H*_*ex*_ and the C:N ratio of fish muscle tissue (Fig 1), indicating that tissues with higher lipid content (C:N ratio) have less *H*_*ex*_. Zooplankton had a similar negative correlation between C:N and *H*_*ex*_, but due to lower sample size this correlation was not significant (*r* = -0.42, *p* = 0.07, *n* = 19). In contrast, both benthic macroinvertebrates and plants showed no correlation between C:N and the amount of *H*_*ex*_ (Fig 1).

**Table 1:** Fraction of *H*_*ex*_ in different sample types.

Sample	*N*	Min-Max	Mean±SD	Group^a^
Fish muscle	72	0.16−0.36	0.27±0.04	A
Benthic invertebrates	33	0.21−0.36	0.27±0.03	A
Zooplankton	19	0.07−0.30	0.19±0.06	B,C
Aquatic macrophytes	6	0.16−0.30	0.23±0.05	A,B
Terrestrial plants	6	0.09−0.19	0.15±0.04	C
Seston/periphyton	7	0.02−0.12	0.08±0.04	D

^a^Tissues are grouped (A−D) into homogeneous subgroups according to multiple comparisons test (Tukey, α = 0.05).

**Fig 2:**
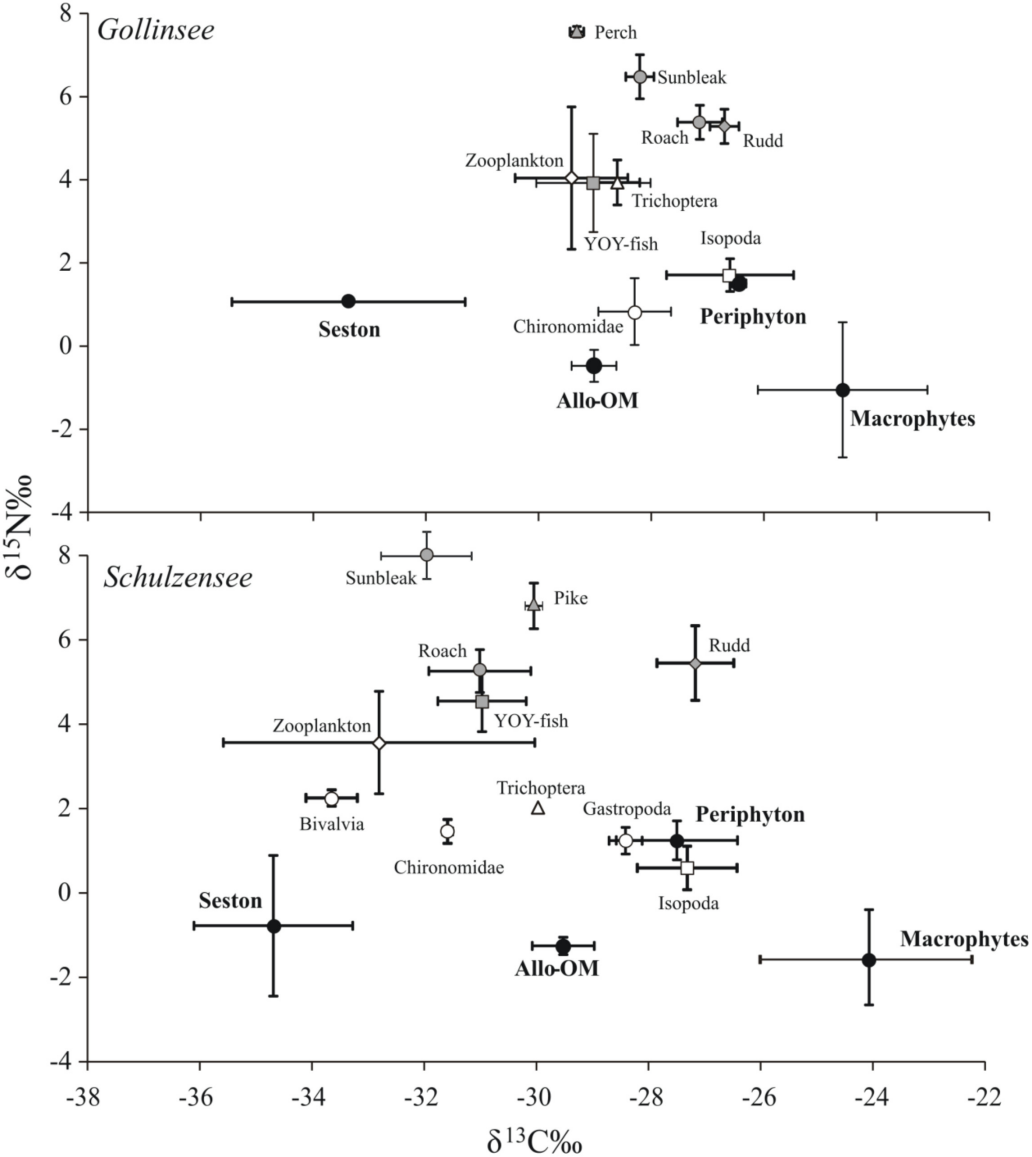
Isotope biplots of δ^13^C and δ^15^N values of the food webs in Gollinsee (upper panel) and Schulzensee (lower panel). The figures illustrate sufficient separation but considerable overlap in δ^13^C and δ^15^N values among the sources in both lakes.

**Fig 3:**
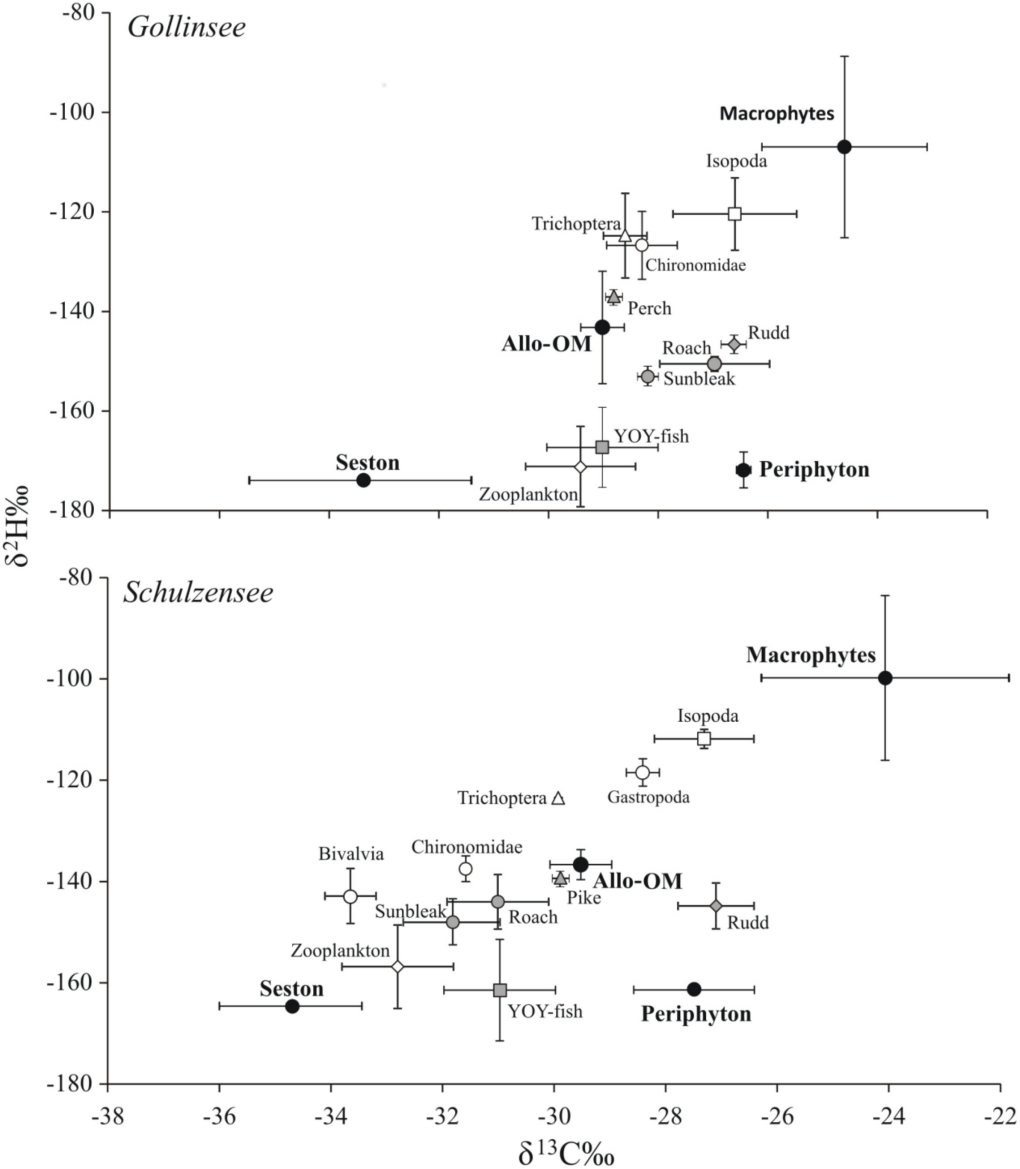
Isotope biplots of δ^2^H and δ^13^C values of the food webs in Gollinsee and Schulzensee. H and C isotopes reveal a distinct separation of sources in both lakes and the typical pattern of low and identical δ^2^H values in pelagic and littoral algal production, higher δ^2^H values in terrestrial organic matter but the highest δ^2^H values in aquatic macrophytes.

Carbon stable isotope values in Gollinsee and Schulzensee food webs varied roughly by 10‰ with lowest δ^13^C values (-33 to -35‰) observed in pelagic seston and highest (-24 to -25‰) in aquatic macrophytes in both lakes (Fig 2). The δ^15^N values similarly varied by almost 10‰ from producers to consumers with slightly higher values in pelagic consumers (zooplankton, planktivorous fish) than in more littoral consumers (benthic macroinvertebrates, benthivorous fish), a pattern often observed in eutrophic lakes (e.g. [58]). The mean isotope values of sources (i.e. seston, periphyton, allo-om and macrophytes) were similar between the two lakes (MANOVA; *p* = 0.438) but differed from each other particularly in δ^13^C and δ^2^H values. In fact, the mean δ^2^H values were clearly distinct and separated (Fig 3), except between seston and periphyton, and δ^2^H clearly had the highest effect size (*F*_*3*,*11*_ = 41.5; *p* < 0.001; partial *η*^*2*^ = 0.93). Mean δ^13^C values also provided a reasonable separation, particularly among seston, allo-om and macrophytes (*F*_*3*,*11*_ = 19.9; *p* < 0.001; partial *η*^*2*^ = 0.84), but could not separate periphyton from allo-om or macrophytes. Only periphyton was separated by its mean δ^15^N values from other sources (*F*_*3*,*11*_ = 6.1; *p* = 0.011; partial *η*^*2*^ = 0.62) and overall the values were strongly overlapping. In general, seston and periphyton had the lowest and nearly identical mean (± SD) δ^2^H values at -161 ± 0.1‰ to -174 ± 3.6‰ with very little variation around the mean values. Allo-om had mean δ^2^H values ~30‰ elevated to seston/periphyton at -143 ± 11‰ in Gollinsee and -137 ± 2‰ in Schulzensee, while the most elevated mean δ^2^H values were observed in aquatic macrophytes (-107 ± 18‰ in Gollinsee and -100 ± 16‰ in Schulzensee). δ^2^H values were not significantly different among submerged (*C*. *submersum*), floating-leaved (*N*. *alba*) and emerged (reed) macrophytes.

Consumers in both lakes were positioned as expected within the δ^13^C-δ^15^N-δ^2^H space, after accounting for the environmental water on consumer H, which in Gollinsee had a mean δ^2^H value of -33 ± 0.3‰ and in Schulzensee -28 ± 0.6‰. The more pelagic consumers were clearly aligned towards seston δ^13^C and δ^2^H values while the more littoral benthic invertebrates had higher values and some clearly approached the values of aquatic macrophytes (Fig 3). Rudd, a known herbivorous fish [59], differed from other fish by its isotope values, particularly in macrophyte-rich Schulzensee (Figs 2 and 3), having isotope values indicative of its consumption of macrophytes and periphyton, and the highest estimates of allo-om contribution (Table 2).

**Table 2:** Mean allo-om contributions to consumers in Gollinsee and Schulzensee.

			HCN model		CN model		HCN no macrophytes	
Lake	Taxa	*t*±SD^a^	Mean	SD	Mean	SD	Mean	SD
*Gollinsee*	Zooplankton	2	0.09	0.08	0.21	0.13	0.11	0.08
	Bivalvia	2	0.17	0.14	0.22	0.13	0.24	0.15
	Trichoptera	2	0.24	0.14	0.24	0.14	0.31	0.19
	Chironomidae	2	0.31	0.16	0.29	0.15	0.55	0.22
	Isopoda	2	0.32	0.13	0.33	0.14	0.78	0.19
	YOY-fish	2.5±0.3	0.11	0.12	0.23	0.33	0.15	0.12
	Sunbleak	3.1±0.2	0.16	0.13	0.23	0.24	0.25	0.13
	Rudd	3.0±0.2	0.18	0.15	0.24	0.28	0.28	0.15
	Roach	2.8±0.4	0.17	0.08	0.25	0.20	0.27	0.08
	Perch	3.4±0.0	0.19	0.28	0.24	0.29	0.30	0.28
*Schulzensee*	Zooplankton	2	0.07	0.06	0.14	0.11	0.09	0.07
	Bivalvia	2	0.18	0.14	0.26	0.14	0.46	0.12
	Trichoptera	2	0.26	0.14	0.25	0.14	0.37	0.18
	Chironomidae	2	0.30	0.15	0.28	0.15	0.55	0.19
	Isopoda	2	0.32	0.14	0.30	0.14	0.53	0.23
	Gastropoda	2	0.35	0.16	0.33	0.16	0.71	0.19
	YOY-fish	3.0±0.2	0.13	0.21	0.22	0.33	0.19	0.21
	Sunbleak	3.6±0.1	0.17	0.17	0.18	0.30	0.27	0.17
	Rudd	3.6±0.2	0.21	0.14	0.23	0.27	0.35	0.14
	Roach	3.2±0.4	0.17	0.08	0.22	0.26	0.26	0.08
	Pike	3.8±0.1	0.21	0.21	0.21	0.22	0.35	0.21

The models refer to a three-isotope model (HCN), a two-isotope model (CN) and a three-isotope model without macrophytes as one source (HCN no macrophytes). Standard deviation (SD) for allo-om contribution is calculated from the variance of probability distribution for allo-om source (zooplankton and macroinvertebrates) and from a sum of variances from multiple resources (fish) [54,55].

^a^*t* is the assigned (invertebrates) or estimated (fish) trophic position used to correct for the environmental hydrogen.

**Fig 4:**
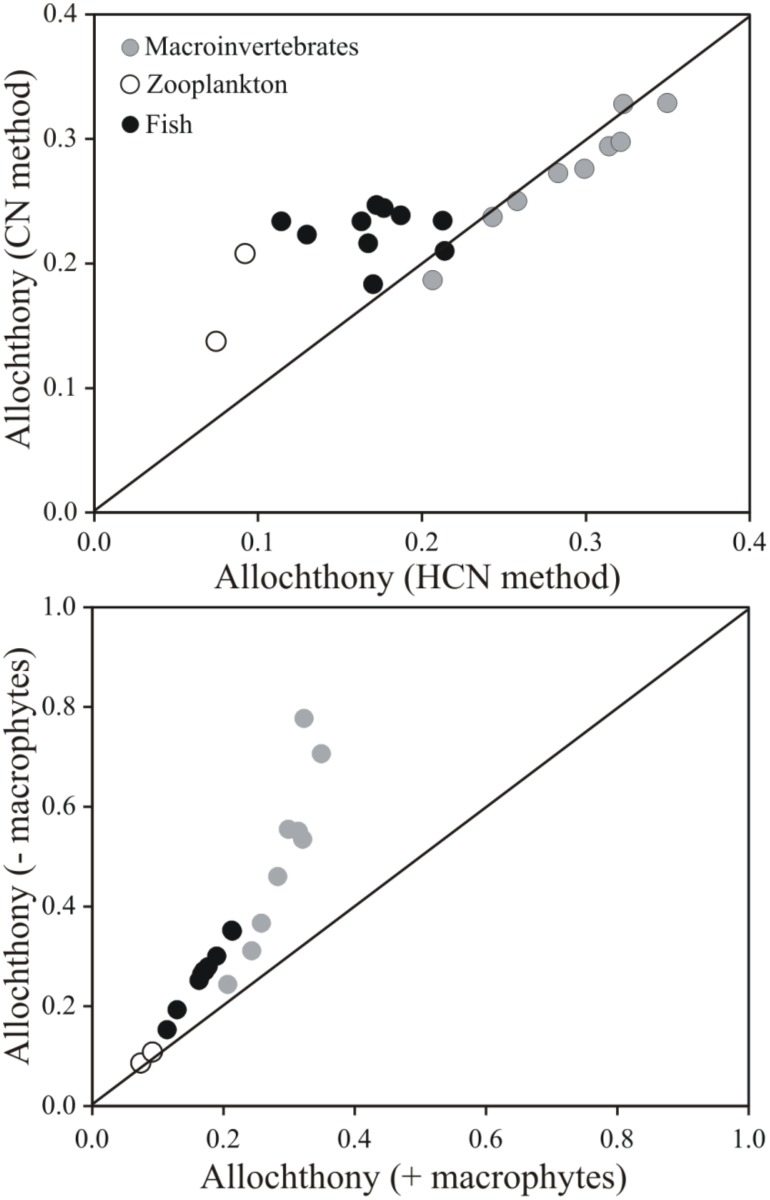
A comparison of consumer allochthony values from different models. Models used either δ^13^C and δ^15^N values or δ^13^C, δ^15^N and δ^2^H values (CN and HCN methods, upper panel) or a HCN mixing model with either macrophytes excluded from the model (y-axis, -macrophytes) or macrophytes included (x-axis, +macrophytes) to estimate consumer allochthony. The lines indicate a 1:1 fit. More detailed values with uncertainties are provided in Table 2.

Calculating mean allochthonous carbon contributions to different consumers in both food webs using either the CN (δ^13^C and δ^15^N) or the HCN (δ^13^C, δ^15^N and δ^2^H) models resulted in small and insignificant differences for the macroinvertebrate consumers (paired t-test; *p* = 0.85, Fig 4 upper panel). Mean allo-om contributions (± SD) to benthic macroinvertebrates in Gollinsee were 27 ± 5% using CN and 26 ± 7% using HCN and equal in Schulzensee at 28 ± 3% with CN and 28 ± 7% with HCN model (Table 2). Though not statistically significant, there was more difference in mean allo-om contribution between the two models within zooplankton samples; 21% and 14% in Gollinsee and Schulzensee using CN but only 9% and 7% using HCN, respectively (Fig 4, Table 2). Full mixing model results and reliance of invertebrate consumers from other carbon sources are provided in a supplementary S1 Table. Using only C and N isotopes resulted in similarly higher estimates of mean allo-om contribution to fish compared to HCN (*t*_*9*_ = -5.37, *p* < 0.001). Using C and N, the mean contribution of allo-om was 24 ± 1% and 21 ± 2% to fish biomass in Gollinsee and Schulzensee whereas using HCN, the values decreased to 16 ± 3% and 18 ± 4%, respectively. Including δ^2^H values in the model also increased the precision of mixing models, particularly at higher trophic levels, by decreasing the variation in observed probability distributions (Table 2).

Comparing the HCN mixing models with and without aquatic macrophytes as one potential source for consumers resulted in more striking differences in allo-om contributions (Fig 4, lower panel, Table 2). While there was little difference in the estimates of allo-om contribution to zooplankton biomass with or without macrophytes, the impacts on macroinvertebrate and thereby fish estimates were significant (*t*_9_ = -2.92, *p* = 0.017 for macroinvertebrates and t_9_ = -2.20, *p* = 0.049 for fish). Excluding macrophytes from the model increased the allo-om contribution to benthic macroinvertebrates to 47 ± 24% (from 26 ± 7%) and 52 ± 13% (28 ± 7%) in Gollinsee and Schulzensee. The highest contribution of allo-om was found in detritivorous *Asellus aquaticus* (Isopoda) in Gollinsee, which approached 80%. For fish, the estimates increased to 29 ± 7% (16 ± 3%) and 25 ± 6% (18 ± 4%), respectively.

## Discussion

This study underlines key issues that require more attention when using stable isotopes of hydrogen with carbon and nitrogen to study the structure and energy flow in freshwater ecosystems. Specifically, studies combining δ^2^H with δ^13^C and δ^15^N to trace allochthonous contributions in lake food webs need to be more aware of the possible δ^2^H values in different sources of organic matter in lakes and therefore carefully consider the choice of end-members (sources) in their mixing models. Most previous studies have not analyzed or reported δ^2^H of macrophytes, but instead used phytoplankton and/or attached algae as sole representatives for δ^2^H of autochthonous production [20, 23, 25–27, 60–61], assuming either insignificant contributions from aquatic macrophytes (or no presence of macrophytes) or equal δ^2^H values for all autochthonous production. Many of these studies assessed the allochthony of zooplankton to which macrophyte-derived organic matter might not significantly contribute independent of their presence in the lake, as shown in this study. However, terrestrial particulate organic matter (POM) has been shown to substantially support zooplankton [6] in more oligotrophic systems and therefore POM from macrophytes could be a significant source for zooplankton in these systems, assuming direct consumption of POM (but see Brett et al. [62]).

Our results show that *i*) macrophytes are an important dietary source for many aquatic consumers and, together with similar findings from previous studies [37–38], also *ii*) indicate that δ^2^H of higher aquatic plants can vary substantially from that of algae, with important implications for estimating allochthonous matter contributions derived from stable isotope mixing models. Further, the experimental equilibration of samples done here under two distinct δ^2^H of water vapor revealed differences in the amount of *H*_*ex*_ in our sample materials which call for further examination.

### *H*_*ex*_ in sample materials

While there was practically no difference between the mean *H*_*ex*_ in fish and benthic macroinvertebrates, both had significantly more *H*_*ex*_ than the zooplankton samples. Similarly, the amount of *H*_*ex*_ was low, but more variable, in all our seston and terrestrial plant samples. It is important to understand the potential implications of varying amount of *H*_*ex*_ in sample materials, particularly when choosing suitable standards for analyses of δ^2^H from organic materials. As the exchangeable part of H in sample tissues is freely exchangeable with the H in water vapor, the standard materials should ideally have similar amounts of *H*_*ex*_ as the samples. The δ^2^H values in air can fluctuate seasonally and differ markedly from those in the samples and therefore both differences in *H*_*ex*_ and variation in δ^2^H of air will introduce bias into the final analysis results.

At present, keratin standards (such as powdered hair, horn, hoof, and whale baleen) are primarily used and available for reliable δ^2^H analyses. While keratin in feathers for example can theoretically have as much as 40% *H*_*ex*_ [18], experimental studies have shown that these materials typically contain less than 20% *H*_*ex*_ [16–17, 36] and biological materials generally contain between 10–30% [36]. Our fish muscle and macroinvertebrate samples contained on average 27% *H*_*ex*_ which is somewhat higher than expected, but within the typical ranges. It is possible that the δ^2^H value used in our calculations (laboratory air moisture -91 ± 8‰, taken from the seasonal average values for precipitation at Flagstaff, Arizona, for the analysis time), is not entirely accurate. But even assuming a more negative value for the same area, such as -110‰ reported in Doucett et al. [20], would reduce the average %*H*_*ex*_ value by only 1% unit for both fish and macroinvertebrates. Zooplankton as well as terrestrial and aquatic plants had on average 15–23% *H*_*ex*_ fitting relatively well with the ~20% *H*_*ex*_ of keratin standards, while seston/periphyton samples had much less *H*_*ex*_, on average just 8%. However, even though the differences in %-values may seem rather high, the impact on the final δ^2^H values may not be as dramatic when considering the expected differences among organic matter sources in fresh water systems, and other uncertainties associated with the mean δ^2^H values (Table 3). A difference <10‰ would probably not have a significant impact in systems with sufficient differences among the source δ^2^H values but needs to be considered when sources are less well separated and the *H*_*ex*_ between the sample and the standard are more considerable (but see discussion in Wilkinson et al. [28]). Based on results presented here, the commonly used standard materials seem appropriate for aquatic consumer tissues but care must be taken when selecting standard materials for producer samples (such as those used here as sources).

**Table 3:** Potential influence of changing δ^2^H in air moisture on δ^2^H values in a sample with variable *H*_*ex*_.

	Air moisture δ^2^H				
	-50‰		-200‰		
Sample *H*_*ex*_	Standard (‰)	Sample (‰)	Standard (‰)	Sample (‰)	Difference (‰)
5%	-82.0	-114.5	-112.0	-122.0	22.5
10%	-82.0	-117.0	-112.0	-132.0	15.0
15%	-82.0	-119.5	-112.0	-142.0	7.5
20%	-82.0	-122.0	-112.0	-152.0	0.0
25%	-82.0	-124.5	-112.0	-162.0	7.5
30%	-82.0	-127.0	-112.0	-172.0	15.0
35%	-82.0	-129.5	-112.0	-182.0	22.5

The standard material is assumed to contain 20% *H*_*ex*_ and the difference indicates the potential bias observed when a sample (with given *H*_*ex*_) and the standard are equilibrated under air moisture δ^2^H of -50‰ and -200‰.

Of greater importance is unquestionably the sample lipid content [22, 32]. Lipids are known to be extremely (-100‰) depleted in ^2^H [34–35] and therefore could strongly influence the observed bulk sample δ^2^H values [32], being analogous to bulk sample lipid content and δ^13^C values [54]. Also, lipids contain very little *H*_*ex*_ [16, 36]; hence samples with high lipid content may have much less *H*_*ex*_ [16, 22, 48] with subsequent implications beyond the immediate effect on δ^2^H values of bulk samples. Using the bulk sample C:N ratio as a proxy for lipid content [56–57], the amount of *H*_*ex*_ correlated strongly with fish muscle tissue and a weak relationship was also evident between zooplankton C:N ratios and *H*_*ex*_, indicating that the samples with higher lipid content had less *H*_*ex*_. Fish and zooplankton can both accumulate appreciable amounts of lipids in their body tissues and our results here demonstrate that in addition to the direct impacts on sample δ^2^H values, this will influence the amount of *H*_*ex*_ in sample materials. However, the C:N ratio does not seem to correlate as strongly with the lipid content in benthic macroinvertebrates or in plant material, likely due to variable amounts of chitin in whole body invertebrate samples [56] and plasticity in nitrogen uptake and allocation in plants [63], which is likely the reason for no correlation between the amount of *H*_*ex*_ and these sample materials here. It is strongly recommended that future food web studies using δ^2^H values either extract lipids from their samples (see further recommendations in Soto et al. [32] and Wilkinson et al. [28]) or work towards developing suitable δ^2^H normalization models similar to δ^13^C based on sample lipid content.

### δ^2^H in primary producers

As expected, the δ^2^H values of water from both studied lakes were similar and fit well into the global isoscapes of δ^2^H in precipitation for North-Eastern Germany [64] and the observed values in precipitation for summer months in this region (www.waterisotopes.org). These values were therefore considered to represent δ^2^H in lake water for the entire summer. The δ^2^H values in pelagic phytoplankton (or seston) and attached algae/periphyton were ~130‰ more negative than the surrounding water, a value very similar to those found in many other studies [20, 23, 26, 38]. However, terrestrial vegetation (alder and birch leaves) had δ^2^H values only ~30‰ elevated from those of algae creating far less separation between δ^2^H values of allochthonous organic matter and algae than reported for example from more arid environments [20], or even from boreal humic lakes of northern Sweden [23]. Rather surprisingly, the macrophytes in our lakes had even higher δ^2^H values than terrestrial vegetation, being roughly another 30‰ enriched in ^2^H and we observed no differences in δ^2^H values among submerged, floating-leaved and emerged macrophytes. While surprising, a few other studies have also reported unexpectedly high δ^2^H values for macrophytes [37–38]. Photosynthesis is assumed to involve a discrimination in δ^2^H, making plants 160–170‰ more negative in their δ^2^H values than the environmental water [38, and references therein] and terrestrial plants with an additional transpiration process are expected to be considerably elevated in their final δ^2^H values compared to aquatic plants. At present it is difficult to explain the observed δ^2^H patterns in aquatic vegetation (but see discussion in [38]), but the distinct δ^2^H values of macrophytes in our lakes helped to verify their importance for lake consumers. The δ^13^C values often overlap among benthic algae, macrophytes and terrestrial sources, while the δ^15^N can be highly variable in aquatic systems [56, 65] due to the many potential sources of inorganic N [66], making these less efficient in separating multiple sources for mixing models.

### Estimates of allochthonous contributions

The importance of including macrophytes as one potential source of organic matter in the mixing models when estimating allochthonous contributions was highlighted in the comparison of models with and without macrophytes (Fig 4, lower panel). While the difference in the estimates of allochthony for zooplankton was minimal between the two models, including macrophytes considerably reduced allochthony in fish, and particularly in benthic macroinvertebrates. Without macrophytes, allochthony of the isopod *A*. *aquaticus* and gastropods was as high as 70–80%, similar to the very high contributions reported in some previous studies [6, 67]. However, allochthony declined to 32–35% when macrophytes were added to the model, confirming the importance of macrophytes as one source of organic matter to consumers. In fact, Brothers et al. [45–46] made a detailed study on C fluxes in these lakes, including C flux from reeds and other macrophytes. Detailed measurements of macrophyte areas, densities and biomass with direct measurements and literature data of C content revealed high C influx from reeds and other macrophytes in both lakes (86% of C influx in Gollinsee i.e. 214 g C m^-2^ yr^-1^ and 72% i.e. 119 g C m^-2^ yr^-1^ in Schulzensee) [45, 54], indicating high autochthonous production. Therefore the lower estimates of allochthony in this study seem more realistic for these lakes. The importance of this finding is highlighted by the fact that in many studies using H (HC/HCN) isotopes only algae or periphyton are used as autochthonous mixing model end-member (20, 22–25, 61). While primary production in many lakes may indeed be dominated by algae, and macrophyte and reed abundance is low, in other lakes (such as those studied here) macrophytes contribute substantially to primary production [45]. Our calculations illustrate the potential implications of excluding macrophytes in the mixing models when they are present. As the macrophyte δ^2^H values in both our lakes were considerably elevated compared to other sources of organic matter, even rather small amounts of organic matter derived from macrophytes can significantly bias the estimates, if only algae and terrestrial matter were assumed as sources. Therefore, macrophytes should be included as one source in mixing models for lakes where they are present (including reeds in the littoral).

Estimates of consumer allochthony using either CN or HCN mixing models (Fig 4, upper panel) were more similar than those comparing HCN model with and without macrophytes. In fact, for benthic macroinvertebrates the CN and HCN models resulted in almost equal estimates of allochthony. In contrast, fish and particularly zooplankton allochthony were estimated higher using the traditional CN model but the difference could partly relate to the impacts of lipids on consumer δ^2^H values. Since lipids have very low δ^2^H values, even small amounts of storage lipids can have significant impact on the bulk sample δ^2^H values. We did not remove lipids from our samples but in particular zooplankton is known to accumulate appreciable amounts of lipids, considerably affecting the isotope values analyzed from bulk samples [57]. Therefore, removing lipids would certainly have elevated the δ^2^H values of zooplankton and would have increased the allochthony in HCN mixing models. In addition, very little is known about the potential discrimination of H isotopes in trophic transfers from prey to consumers. In our mixing models we used the common practice of assuming no real trophic discrimination, but did assume ~17% contribution from environmental water at each trophic level [30]. While our results do not point to any major additional trophic discrimination, these assumptions can influence the outcome particularly at the higher trophic levels [28] and therefore more laboratory work is urgently needed to verify the robustness of these assumptions.

## Conclusions

Our results highlight the benefits of combining δ^2^H values with δ^13^C and δ^15^N in food web studies, particularly when multiple resources need to be separated. However, our comparisons of multiple sample materials for differences in *H*_*ex*_ revealed significantly higher proportions of *H*_*ex*_ in animal tissues compared to plant tissues (excluding macrophytes). In general, however, the results fit to expected ranges relatively well and align with those of most common standard materials (such as keratin). Nonetheless, we concur with previous studies [28, 32–33] urging for greater caution when interpreting δ^2^H results before more laboratory and field studies on the effects of environmental water, lipids and δ^2^H patterns in ecosystems are available. Furthermore, the importance of aquatic macrophytes as a dietary source for lake consumers was detected more clearly when using δ^2^H. Due to the high δ^2^H values of macrophytes, estimates of allochthony by mixing models were largely dependent on whether macrophytes were included or not. We therefore strongly recommend future studies to include macrophytes as a potential resource when using δ^2^H with δ^13^C and δ^15^N in aquatic food web studies.

## Supporting Information

S1 Dataset Stable HCN isotope data used in this study. (XLSX)

S1 Table Mixing model source contributions to invertebrate consumers in Gollinsee and Schulzensee. (PDF)
