# Supplementary material for: Assessing the Utility of Hydrogen, Carbon and Nitrogen Stable Isotopes in Estimating Consumer Allochthony in Two Shallow Eutrophic Lakes
Source: PLoS One. 2016 May 11;11(5):e0155562. doi: 10.1371/journal.pone.0155562 (PMC4863965; doi:10.1371/journal.pone.0155562)
Supplement: S1 Table — (PDF) [file pone.0155562.s002.pdf]

S1 Table. Mean source contributions to invertebrate consumers in Gollinsee and Schulzensee using different mixing models. The models refer to a three-isotope model (HCN), a two-isotope model (CN) and a three-isotope model without macrophytes as one source (HCN – no macrophytes). Low and high 95% are credibility intervals for SIAR output mean values.

			HCN model			CN model			HCN - no macrophytes		
Lake	Taxa	Source	Mean	Low 95%	High 95%	Mean	Low 95%	High 95%	Mean	Low 95%	High 95%
Gollinsee											
	Zooplankton	Allo-om	0.09	0.00	0.25	0.21	0.00	0.43	0.11	0.00	0.28
		Macrophytes	0.04	0.00	0.11	0.12	0.00	0.29			
		Seston	0.46	0.31	0.61	0.42	0.25	0.58	0.45	0.31	0.60
		Periphyton	0.41	0.23	0.58	0.26	0.01	0.47	0.44	0.26	0.60
	Bivalvia	Allo-om	0.17	0.00	0.42	0.19	0.00	0.42	0.24	0.00	0.51
		Macrophytes	0.17	0.00	0.44	0.12	0.00	0.33			
		Seston	0.45	0.04	0.76	0.48	0.12	0.82	0.53	0.19	0.86
		Periphyton	0.21	0.00	0.46	0.22	0.00	0.47	0.22	0.00	0.48
	Trichoptera	Allo-om	0.24	0.00	0.49	0.24	0.00	0.48	0.31	0.00	0.67
		Macrophytes	0.22	0.00	0.45	0.18	0.00	0.39			
		Seston	0.29	0.04	0.49	0.30	0.06	0.51	0.29	0.01	0.53
		Periphyton	0.25	0.00	0.50	0.28	0.00	0.53	0.40	0.08	0.70
	Chironomidae	Allo-om	0.31	0.00	0.59	0.29	0.00	0.56	0.55	0.17	0.97
		Macrophytes	0.34	0.07	0.59	0.24	0.01	0.45			
		Seston	0.23	0.01	0.40	0.26	0.05	0.44	0.15	0.00	0.36
		Periphyton	0.12	0.00	0.33	0.21	0.00	0.42	0.29	0.00	0.54
	Isopoda	Allo-om	0.32	0.04	0.56	0.33	0.04	0.58	0.78	0.41	1.01
		Macrophytes	0.51	0.33	0.69	0.42	0.22	0.63			
		Seston	0.09	0.00	0.22	0.10	0.00	0.25	0.06	0.00	0.18
		Periphyton	0.08	0.00	0.21	0.15	0.00	0.34	0.16	0.00	0.52
Schulzensee											
	Zooplankton	Allo-om	0.07	0.00	0.19	0.14	0.00	0.37	0.09	0.00	0.21
		Macrophytes	0.03	0.00	0.08	0.05	0.00	0.16			
		Seston	0.65	0.36	0.92	0.56	0.25	0.89	0.65	0.37	0.93
		Periphyton	0.24	0.00	0.52	0.25	0.00	0.51	0.26	0.00	0.53
	Bivalvia	Allo-om	0.18	0.01	0.52	0.27	0.00	0.51	0.46	0.21	0.68
		Macrophytes	0.12	0.00	0.26	0.15	0.00	0.33			
		Seston	0.40	0.10	0.65	0.29	0.00	0.57	0.29	0.00	0.56
		Periphyton	0.30	0.02	0.53	0.29	0.01	0.51	0.25	0.00	0.48
	Trichoptera	Allo-om	0.26	0.00	0.50	0.25	0.00	0.49	0.37	0.00	0.70
		Macrophytes	0.24	0.00	0.46	0.24	0.00	0.47			
		Seston	0.26	0.00	0.48	0.25	0.00	0.48	0.32	0.00	0.62
		Periphyton	0.24	0.00	0.46	0.26	0.00	0.49	0.31	0.00	0.60
	Chironomidae	Allo-om	0.30	0.00	0.58	0.28	0.00	0.52	0.55	0.17	0.87
		Macrophytes	0.20	0.01	0.37	0.22	0.00	0.43			
		Seston	0.28	0.01	0.52	0.29	0.00	0.57	0.29	0.00	0.58
		Periphyton	0.22	0.00	0.42	0.22	0.00	0.43	0.15	0.00	0.39
	Isopoda	Allo-om	0.32	0.02	0.57	0.30	0.01	0.55	0.53	0.14	0.97
		Macrophytes	0.44	0.26	0.62	0.37	0.18	0.57			
		Seston	0.12	0.00	0.29	0.16	0.00	0.34	0.14	0.00	0.42
		Periphyton	0.12	0.00	0.29	0.17	0.00	0.38	0.32	0.00	0.69
	Gastropoda	Allo-om	0.35	0.03	0.66	0.33	0.02	0.65	0.71	0.35	0.99
		Macrophytes	0.30	0.10	0.49	0.25	0.06	0.43			
		Seston	0.19	0.00	0.36	0.22	0.01	0.39	0.07	0.00	0.22
		Periphyton	0.15	0.00	0.32	0.20	0.00	0.38	0.22	0.00	0.50
